# A phase II study of NK012, a polymeric micelle formulation of SN-38, in unresectable, metastatic or recurrent colorectal cancer patients

**DOI:** 10.1007/s00280-018-3693-6

**Published:** 2018-10-04

**Authors:** Tetsuya Hamaguchi, Akihito Tsuji, Kensei Yamaguchi, Koji Takeda, Hiroyuki Uetake, Taito Esaki, Kenji Amagai, Daisuke Sakai, Hideo Baba, Masami Kimura, Yasuhiro Matsumura, Tetsuji Tsukamoto

**Affiliations:** 1 Gastrointestinal Medical Oncology Division, National Cancer Center Hospital, Tokyo, Japan; 2 Department of Gastroenterological Oncology, Saitama Medical University International Medical Center, 1397-1 Yamane, Hidaka, Saitama Japan; 3 Division of Medical Oncology, Kochi Health Science Center, Kochi, Japan; 4 Department of Clinical Oncology, Kagawa University Faculty of Medicine Cancer Center, Kagawa University Hospital, Kagawa, Japan; 5 Department of Gastroenterology, Saitama Cancer Center, Saitama, Japan; 6 Department of Gastroenterological Chemotherapy Center, Institute Hospital of Japanese Foundation for Cancer Research, Tokyo, Japan; 7 Departmentof Medical Oncology, Osaka City General Hospital, Osaka, Japan; 8 Approved Specified Nonprofit Corporation West Japan Oncology Group, Osaka, Japan; 9 Department of Specialized Surgeries, Tokyo Medical and Dental University, Tokyo, Japan; 10 Department of Gastrointestinal and Medical Oncology, National Kyushu Cancer Center, Fukuoka, Japan; 11 Department of Gastroenterology, Ibaraki Prefectural Central Hospital, Ibaraki, Japan; 12 Department of Medical Oncology, Osaka International Cancer Institute, Osaka, Japan; 13 Department of Frontier Science for Cancer and Chemotherapy, Graduate School of Medicine, Osaka University, Osaka, Japan; 14 Department of Gastroenterological Surgery, Graduate School of Medical Sciences, Kumamoto University, Kumamoto, Japan; 15 Department of Surgery, Japan Community Health Care Organization, Hitoyoshi Medical Center, Kumamoto, Japan; 16 Division of Developmental Therapeutics, Exploratory Oncology Research and Clinical Trial Center, National Cancer Center, Chiba, Japan; 17 Nippon Kayaku Co., Ltd., Tokyo, Japan

**Keywords:** NK012, Polymeric micelle, SN-38, Colorectal cancer, Phase II

## Abstract

**Purpose:**

NK012 is a polymeric micelle formulation of SN-38, the active metabolite of irinotecan. We evaluated the efficacy and safety of NK012 in Japanese patients with unresectable metastatic colorectal cancer.

**Methods:**

We conducted a multicenter open-label phase II trial of NK012 monotherapy in 58 patients who had been treated with an oxaliplatin-based chemotherapy regimen (group A: 53 patients with *UGT1A1* genotype *–*/*–*, *6/*–*, or *28/*–*; group B: 5 patients with *UGT1A1* genotype *6/*28 or *6/*6). The primary endpoint was the response rate (RR). Initial doses of 28 and 18 mg/m^2^ for group A and group B, respectively, were administered intravenously over 30 min, and these doses were subsequently administered every 3 weeks. Group A was evaluated as the primary efficacy population, while group B was evaluated for reference.

**Results:**

In group A, the RR was 3.8%, and the median progression-free survival and overall survival were 3.30 months and 15.03 months, respectively. In both groups, the most common grade ≥ 3 adverse drug reaction (ADR) was neutropenia and the incidence of grade ≥ 3 diarrhea was low or zero. In group A, 17 serious ADRs were observed in 10 patients (17%); all improved or recovered. In group B, no serious ADRs were observed. No treatment-related deaths were reported in either group.

**Conclusions:**

NK012 monotherapy yielded an RR similar to the RR of irinotecan monotherapy that was reported in the phase III EPIC trial (4.2%), and the incidence of grade ≥ 3 diarrhea was low. Based on the incidence and severity of febrile neutropenia and grade ≥ 3 neutropenia, the initial dose of NK012 28 mg/m^2^ may be too high for colorectal cancer patients who have previously been treated with an oxaliplatin-based chemotherapy regimen.

## Introduction

The number of patients with colorectal cancer has been rapidly increasing worldwide in recent decades. In Japan, in 2012, a total of 134,575 individuals (77,365 men and 57,210 women) received diagnoses of colorectal cancer [1], and in 2015, 49,699 people (26,818 men and 22,881 women) died of colorectal cancer [1].

Since the approval of bevacizumab by the FDA in 2004, remarkable progress has been made in chemotherapy for unresectable metastatic colorectal cancer: the median survival time has exceeded 30 months in the latest clinical trials. The importance of the “continuum of care,” characterized by the strategic and continued use of effective drugs, is widely accepted for the treatment of colorectal cancer.

For the treatment of unresectable metastatic colorectal cancer, the National Comprehensive Cancer Network (NCCN) guidelines recommend the use of fluorinated pyrimidines (+ folinate), oxaliplatin, and irinotecan hydrochloride (CPT-11), either as monotherapy or in combination, as well as add-on bevacizumab, aflibercept and ramucirumab therapies. In patients with wild-type *RAS* gene tumors, treatment with cetuximab or panitumumab is also recommended [2]. When patients have received an oxaliplatin-based combination therapy (e.g., FOLFOX) as their initial therapy, the recommended second-line therapy is a CPT-11-based combination therapy (e.g., FOLFIRI) or CPT-11 monotherapy. By contrast, when patients have received a CPT-11-based combination therapy as their initial therapy, the recommended second-line therapy is an oxaliplatin-based combination therapy. In the case of wild-type *RAS* gene tumors, cetuximab or panitumumab as monotherapy or in combination with CPT-11 is also recommended [2]. CPT-11 has thus been found to be effective in various chemotherapy regimens for metastatic colorectal cancer. Although the overall survival of patients with unresectable metastatic colorectal cancer has been progressively extended with advances in chemotherapy, including the effective use of CPT-11 [3], further improvements in efficacy and safety are required. CPT-11 therapy frequently results in dose-limiting toxicity characterized by neutropenia and diarrhea.

NK012 is a polymeric micelle formulation containing the active metabolite of CPT-11, 7-ethyl-10-hydroxy-camptothecin (SN-38). While both CPT-11 and NK012 contain the same active ingredient (SN-38), NK012, unlike CPT-11, releases SN-38 via hydrolysis, and thus does not require metabolic conversion by enzymes [4]. NK012 is a promising agent for clinical applications given that its ability to suppress tumor cell growth in vitro and its antitumor effects in animal models are stronger than those of CPT-11 in various cell types, including several human tumors. In addition, NK012 has been found to accumulate in high concentrations in tumors in in vivo mouse models [4–8]. Furthermore, NK012 may result in less severe diarrhea than CPT-11, because a higher level of CPT-11 was found in the intestinal lumen compared with NK012 in a preclinical study [9].

In Japan, a phase I clinical trial has been conducted, in which NK012 was administered once every 3 weeks, to investigate the safety and pharmacokinetics of NK012, as well as to determine the recommended dose for a phase II trial [10]. The active ingredient of NK012, SN-38, is metabolized by UGT1A enzymes, and polymorphisms of the *UGT1A1* gene are known to be associated with an increased incidence of severe neutropenia [11]. Therefore, in the phase I trial, the recommended dose was examined mainly in patients who were not homozygous for UGT1A1*28 or UGT1A1*6 or heterozygous for *UGT1A1* *28 and UGT1A1*6. The major dose-limiting toxicity (DLT) was neutropenia, and the recommended dose was determined to be 28 mg/m^2^ [10].

In the above phase I trial, NK012 was administered to a total of 24 patients with malignant tumors, of whom 12 patients had colorectal cancer [10]. Although no patient with colorectal cancer had a partial response (PR), five patients had stable disease (SD). These five patients had a history of treatment with oxaliplatin and CPT-11, and four of them were able to continue to receive the NK012 regimen for ≥ 6 cycles. Moreover, although severe diarrhea often causes discontinuation of CPT-11-based therapy [3], no grade ≥ 3 diarrhea occurred during the phase I trial of NK012. This suggested that NK012 may have a different safety profile than that of CPT-11, and we therefore conducted the present phase II trial to investigate the efficacy and safety of NK012 monotherapy at an initial dose of 28 mg/m^2^ in patients with unresectable metastatic colorectal cancer who had previously received oxaliplatin-based chemotherapy.

## Materials and methods

### Study design and statistical analysis

This multi-center open-label phase II trial of NK012 monotherapy was conducted between June 2009 (first patient on-study) and January 2012 (last patient off-study) at 11 medical institutions in Japan. Patients were registered using a central registration method. Before registration, patients underwent a screening test for *UGT1A1* gene polymorphisms (UGT1A1*6 and UGT1A1*28). Based on the screening results, patients were separated into two groups: group A, which included patients with wild-type (*–*/*–*), UGT1A1*6 heterozygous genotype (*6/*–*), or UGT1A1*28 heterozygous genotype (*28/*–*), and group B, which included patients with UGT1A1*6 homozygous genotype (*6/*6), UGT1A1*28 homozygous genotype (*28/*28), or heterozygous genotype of UGT1A1*6 and UGT1A1*28 (*6/*28). A total of 53 patients were enrolled in group A to determine the efficacy of NK012. In group B, however, a target number of patients was not specified, and efficacy was therefore evaluated for reference purposes in the number of patients recruited.

Tumor specimens collected prior to the current study were tested for *KRAS* exon 2 and 3 gene mutations in an exploratory manner on a voluntary basis during the trial period to determine whether these mutations affect efficacy. The DNA was extracted from paraffin-embedded tumors or surgical frozen tumor samples. The *KRAS* mutation status was assessed by means of direct sequencing by the PCR method.

The primary endpoint was the response rate [complete response (CR) + PR] in group A. The secondary endpoints were the time to treatment failure (TTF), progression-free survival (PFS), overall survival (OS), and adverse events (AE) (for determination of a causal relationship, incidence, and severity). TTF, PFS, and OS were analyzed using the Kaplan–Meier method [12].

Efficacy was evaluated based on the best overall response as adjudicated by independent central review using the new Response Evaluation Criteria in Solid Tumors (RECIST) guidelines (version 1.1) [13]. For the efficacy assessments, tumor lesions were examined before administration and at least once every two cycles during treatment. The efficacy analysis population (full analysis set; FAS) consisted of those patients who were registered in the trial, received NK012 at least once, and met the inclusion criteria. The analysis of efficacy was mainly performed for the patients in group A. In group B, because the number of patients was not specified, efficacy was analyzed for reference purposes for patients recruited during the trial.

Safety was evaluated based on body weight, vital signs, AEs, clinical examinations, and electrocardiography results. AEs were classified and graded using the National Cancer Institute Common Terminology Criteria for Adverse Events (CTCAE) (version 3.0) [14]. ADRs were defined as AEs that were determined to have a causal relationship with the study drug. The safety analysis set (SAS) consisted of patients who received NK012 at least once.

The target number of patients in group A was calculated using Fleming’s single-stage procedure [15] with a threshold response rate of 5%, an expected response rate of 15%, a significance level of 0.05, and a statistical power of 0.8; as a result, the target number for group A was determined to be 53 patients.

The present study was approved by the local institutional review boards and conducted in accordance with the International Conference on Harmonization guidelines for Good Clinical Practice, all applicable regulatory requirements, the protocol, and the guiding principles of the Declaration of Helsinki. The study was registered at Japan Pharmaceutical Information Center, registration number JapicCTI-090780.

### Patients

The present study enrolled patients with histologically or cytologically confirmed colorectal cancer who had progression or relapse after receiving one prior oxaliplatin-based chemotherapy regimen (e.g., FOLFOX) or who could not continue receiving their previous oxaliplatin-based regimens because of adverse drug reactions (ADRs). The other major inclusion criteria were as follows: Eastern Cooperative Oncology Group (ECOG; Zubrod) performance status of 0–2; age between ≥ 20 and < 75 years; life expectancy > 8 weeks; and adequate bone marrow, hepatic, and pulmonary function within 1 week before treatment initiation [absolute neutrophil count > 2000/µl, platelet count > 100,000/µl, hemoglobin > 9 g/dl, total bilirubin < 1.5 mg/dl, aspartate aminotransferase (AST) and alanine aminotransferase (ALT) < 2.5 times the upper limit of normal, creatinine < 1.5 mg/dl, PaO_2_ > 60 mmHg]. Treatment with radiotherapy or chemotherapy must have ceased at least 4 weeks before treatment initiation. Patients were excluded if they had received topoisomerase I inhibitors (e.g., CPT-11 or other investigational drugs), if they had brain metastases that were symptomatic or required treatment, or if they had been diagnosed as having interstitial pneumonia or pulmonary fibrosis by diagnostic imaging. All patients gave written informed consent prior to enrollment in the study.

### Treatments

NK012 was administered intravenously over approximately 30 min, followed by a 3-week rest period; this was considered 1 cycle, and administration was repeated for several cycles until progression of the tumor was observed or the criteria for treatment discontinuation were met. The initial dose was 28 mg/m^2^ for group A and 18 mg/m^2^ for group B. In group B, the dose could be increased according to the dose escalation plan at the discretion of the investigator to up to 28 mg/m^2^ if the patient had grade 2 or lower ADRs. The dose reduction criteria were defined as grade 4 neutropenia that continued for ≥ 1 week, grade 4 thrombocytopenia, and grade ≥ 3 non-hematological ADRs (excluding hypersensitivity, grade 3 decreased appetite, nausea, and vomiting).

## Results

### Patient disposition and characteristics

Table 1 shows the baseline characteristics of the FAS population. In group A (*n* = 53), 39 (73.6%) of the patients were men and 14 (26.4%) were women, and the median age was 62.0 (range 43–73) years. The primary site of cancer was the colon in 52.8% and the rectum in 47.2%. The majority of patients (96.2%) had no history of radiotherapy. The highest proportion of patients (49.1%) had received prior oxaliplatin-based treatment for ≥ 6 months, followed by the proportion who had received it for 3–6 months (39.6%). In group B, all five patients were women, and the median age was 66.0 (range 62–68) years. The primary site of cancer was the colon in three patients (60.0%) and the rectum in two patients (40.0%). No patient received radiotherapy. The number of patients who had received prior oxaliplatin-based treatment for ≥ 6 months and 3–6 months were two patients (40.0%) each. Of the five patients in group B, four patients were heterozygous for *UGT1A1* *28 and UGT1A1*6 (*6/*28), and one patient was homozygous for UGT1A1*6 (*6/*6).

**Table 1:** Baseline characteristics of the study patients (full analysis set/safety analysis set)

	Group A (*n* = 53)	Group B (*n* = 5)
Sex		
Male	39 (73.6)	0
Female	14 (26.4)	5 (100.0)
Age (years)		
Median (range)	62.0 (43–73)	66.0 (62–68)
ECOG performance status		
0	35 (66.0)	2 (40.0)
1	18 (34.0)	3 (60.0)
Primary site of the cancer		
Colon	28 (52.8)	3 (60.0)
Rectum	25 (47.2)	2 (40.0)
Site of metastasis		
Liver	30 (56.6)	5 (100.0)
Lung	23 (43.4)	0
LN	7 (13.2)	1 (80.0)
Others	9 (17.0)	0
*UGT1A1* genotype		
–/–	26 (49.1)	0
*6/–	15 (28.3)	0
*28/–	12 (22.6)	0
*6/*28	0	4 (80.0)
*6/*6	0	1 (20.0)
*28/*28	0	0
*KRAS* codon 2, 3 gene		
Wild-type	32 (60.4)	2 (40.0)
Mutant-type	16 (30.2)	3 (60.0)
Unknown	5 (9.4)	0
Prior radiation therapy		
No	51 (96.2)	5 (100.0)
Yes	2 (3.8)	0
Duration of prior treatment with oxaliplatin		
< 1 month	1 (1.9)	0
≥ 1 month, < 3 months	5 (9.4)	1 (20.0)
≥ 3 months, < 6 months	21 (39.6)	2 (40.0)
≥ 6 months	26 (49.1)	2 (40.0)
Median (range)^a^	177.0 (2–682)	164.0 (45–291)

Data are expressed as n (%) unless otherwise indicated

*ECOG* Eastern Cooperative Oncology Group, *LN* lymph nodes

^a^Days

### Extent of exposure to the study drug

In group A, 53 patients received NK012, as did all five patients in group B. The median number of NK012 treatment cycles was three (range 1–17) in group A and two (range 1–9) in group B.

Of the 53 patients in group A, 5 patients discontinued treatment at cycle 1. Reasons for discontinuation during cycle 1 were as follows: disease progression in two cases, investigator’s discretion in two cases, and patient’s request in one case. Of the 48 patients who continued treatment through subsequent cycles, a total of 32 patients (66.7%) had their dose reduced to 22 mg/m^2^; of these, 27 patients had their doses reduced at cycle 2, mainly due to neutropenia with or without febrile neutropenia. The breakdown of these cases of neutropenia was as follows: grade 4 neutropenia lasting 7 days or more in 6 cases, grade 3 febrile neutropenia in 6 cases, both grade 4 neutropenia lasting 7 days or more and grade 3 febrile neutropenia in 4 cases, grade 4 neutropenia lasting fewer than 7 days but requiring dose reduction in the physician’s judgment in 11 cases. Of 28 patients who continued receiving treatment in cycle 3 and later cycles, 10 patients (35.7%) had their doses reduced to < 22 mg/m^2^. In group B, one of the five patients discontinued treatment at cycle 1 due to disease progression. Of the four patients who continued receiving treatment in subsequent cycles, four had their doses reduced to 15 mg/m^2^ at cycle 2, two who experienced grade 4 neutropenia lasting 7 days or more, and two who experienced grade 4 neutropenia lasting fewer than 7 days but whose doses needed to be reduced in the physician’s judgment. Excluding patients who discontinued treatment at cycle 1, 34 of the 48 patients (70.8%) in group A and 3 of the 4 patients in group B postponed treatment. No patients in group B had their doses increased.

### Efficacy

Table 2 shows the best overall response in the FAS based on independent central review. In group A, all 53 patients were included in the efficacy analysis; no patient had CR, 2 patients had PR, and 28 patients had SD. The response rate was 3.8% [95% confidential interval (CI) 0.5–13.0], and the disease control rate (DCR: CR + PR + SD) was 56.6% (95% CI 42.3–70.2). The median response duration among the 3 patients with PR (including 1 patient with unconfirmed PR) was 141 (range 35–183) days. In five patients in group B, no patient had CR or PR, and one patient had SD.

**Table 2:** Best overall response (group A, full analysis set)

Number of patients with					Response^a^ rate (95% confidence interval), %	Disease control^b^ rate (95% confidence interval), %
CR	PR	SD	PD	NE		
0	2	28	20	3	3.8 (0.5–13.0)	56.6 (42.3–70.2)

*CR* complete response, *PR* partial response, *SD* stable disease, *PD* progressive disease, *NE* not evaluable

^a^CR + PR

^b^CR + PR + SD

**Fig. 1:**
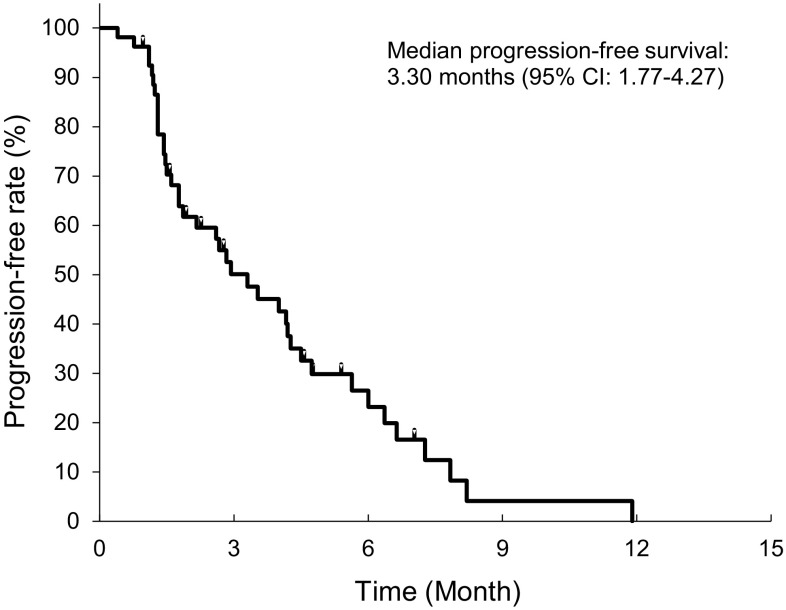
Kaplan–Meier curve for progression-free survival in group A (*N* = 53). The total number of events and the number of censored events are 40 and 13, respectively

**Fig. 2:**
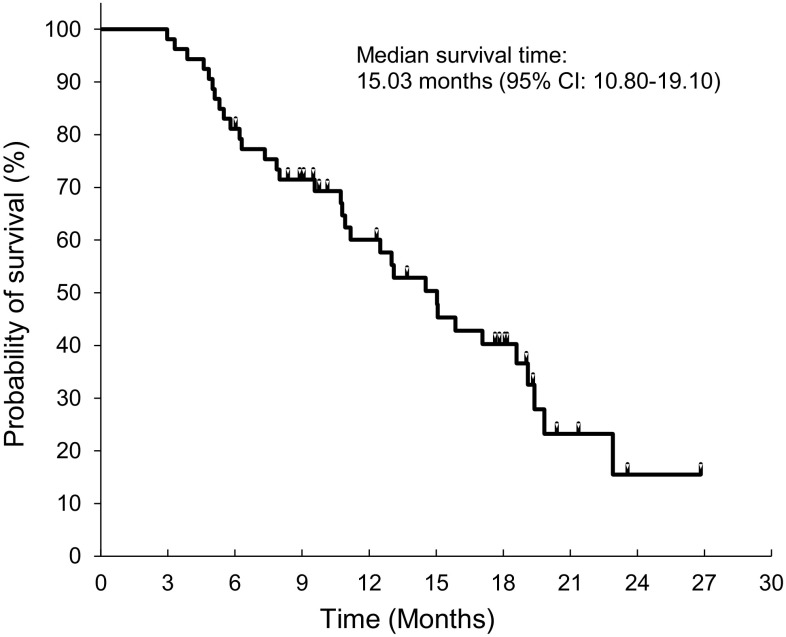
Kaplan–Meier curve for overall survival in the group A (*N* = 53). The total number of events and the number of censored events are 33 and 20, respectively

The median TTF and PFS in group A were 2.83 months (95% CI 1.77–4.20) and 3.30 months (95% CI 1.77–4.27), respectively (Fig. 1). PFS was ≥ 6 months in 8 patients (23.2% estimated on the basis of a Kaplan–Meier curve for PFS). The median OS was 15.03 months (95% CI 10.80–19.10) (Fig. 2).

In a subgroup analysis of group A, because the response rate was low, the DCR was mainly analyzed. The DCR in patients who had target lesions in the lungs (23 patients) was 78.3% (95% CI 56.3–92.5), whereas the DCR for those without target lesions in the lungs (30 patients) was 40.0% (95% CI 22.7–59.4). No difference in the DCR was noted between patients with different *UGT1A1* gene polymorphisms or between patients with and without *KRAS* mutations.

### Safety

Table 3 shows the ADRs that occurred in ≥ 30% of the 53 patients in group A (any grade and grade ≥ 3). Alopecia was the most commonly observed (96.2%) ADR classified as a subjective or objective symptom. Other subjective or objective symptoms occurring in > 50% of subjects included decreased appetite, fatigue, diarrhea, and nausea. No subjective or objective symptom was reported as a grade ≥ 4 ADR. The most common major grade ≥ 3 ADR was febrile neutropenia (26.4%). The incidence of grade 3 diarrhea was 5.7%.

**Table 3:** Adverse drug reactions which occurred in > 30% of patients in group A (*n* = 53) (any grade and grade ≥ 3)

	Group A			Group B		
	Any grade	Grade 3	Grade 4	Any grade	Grade 3	Grade 4
Hematological toxicities						
Neutropenia	53 (100.0)	9 (17.0)	43 (81.1)	5 (100.0)	1 (20)	3 (60)
Leucopenia	53 (100.0)	30 (56.6)	16 (30.2)	5 (100.0)	3 (60)	
Lymphocytopenia	42 (79.2)	14 (26.4)	1 (1.9)	3 (60.0)	2 (40)	
Hemoglobin decreased	39 (73.6)	2 (3.8)	1 (1.9)	3 (60.0)		
Thrombocytopenia	34 (64.2)	5 (9.4)	4 (7.5)	2 (40.0)		
Non-hematological toxicities						
Alopecia	51 (96.2)			5 (100.0)		
Decreased appetite	45 (84.9)	8 (15.1)		3 (60.0)	1 (20.0)	
Fatigue	44 (83.0)	8 (15.1)		3 (60.0)		
Diarrhea	35 (66.0)	3 (5.7)		2 (40.0)		
Nausea	33 (62.3)	2 (3.8)		4 (80.0)	1 (20.0)	
Blood albumin decreased	26 (49.1)	1 (1.9)		3 (60.0)		
Alanine aminotransferase increased	23 (43.4)	1 (1.9)				
Gamma-glutamyltransferase increased	22 (41.5)	7 (13.2)				
Aspartate aminotransferase increased	18 (34.0)	2 (3.8)				

Data are expressed as *n* (%)

Treatment-related adverse events

Neutropenia occurred in all treated patients; grade ≥ 3 neutropenia was observed in 98.1% of the subjects in group A, but most of them recovered. The proportion of courses in which granulocyte colony-stimulating factor (G-CSF) was used was 24.2% (55/227 courses). The incidence of grade 4 neutropenia (< 500 cells/µl) was higher in patients with longer oxaliplatin-based treatment durations [≥ 6 months, 88.5% (23/26 patients); 3–6 months, 76.2% (16/21 patients); < 3 months, 66.7% (4/6 patients)].

Although the number of patients enrolled in group B was small (*n* = 5), grade ≥ 3 ADRs were observed, including neutropenia (4 patients, 80%), leucopenia (3 patients, 60%), decreased appetite (1 patient, 20%), and nausea (1 patient, 20%). The only grade 3 ADR that was observed in group B but not listed in Table 3 (because the incidence was < 30% in group A) was decreased hematocrit. The proportion of courses in which G-CSF was used was 12.5% (2/16 courses).

No treatment-related deaths occurred in either group A or group B. The following serious ADRs were reported in group A: seven events of febrile neutropenia, four events of decreased appetite, and one event each of fatigue, paralytic ileus, nausea, cognitive impairment, neutropenia, and decreased platelet count (a total of 17 events in 10 patients). In each case, the reason for classification as a serious ADR was hospitalization for treatment. All of these serious ADRs improved or recovered after appropriate measures, including medication, were administered. In group B, no serious ADRs were reported.

## Discussion

CPT-11 is a key drug in various chemotherapy regimens for unresectable metastatic colorectal cancer, such as FOLFIRI combination chemotherapy, add-on therapy with cetuximab or panitumumab, or monotherapy. In the EPIC trials, which was a phase III trial of CPT-11 that compared cetuximab plus CPT-11 regimen to CPT-11 monotherapy in a patient population similar to that of the present study, the response rate in patients who received CPT-11 monotherapy (350 mg/m^2^) once every 3 weeks was 4.2%, and the median PFS and OS were 2.6 months and 10.0 months, respectively [16]. The response rate reported in the EPIC trial was similar to that in the present study (3.8% in group A). The median PFS in group A in the present study was 3.30 months, which was slightly longer than that in the EPIC trial. The median OS was also longer in the present study (15.03 months). However, further investigation is necessary into factors such as the number of enrolled patients and the influence of subsequent treatments.

The DCR in group A was 56.6%, which was higher than that in the EPIC trial (45.8%). In the subgroup analysis, the DCR was higher in patients with target lesions in the lungs than in those without (78.3% vs. 40.0%). Similar results were reported in the phase II trial of CPT-11 (DCR 92.9% vs. 7.1%) [17]. As for the relationship between efficacy and *KRAS* status, in the present study, no clear difference was found in the DCR between patients with and without *KRAS* mutations, suggesting that NK012 may be effective regardless of *KRAS* status.

In the present study of NK012 monotherapy, the incidence of grade ≥ 3 fatigue, decreased appetite, neutropenia, and decreased platelet count was 15.1%, 15.1%, 98.1%, and 17%, respectively, in group A. The incidence of these ADRs was higher than those in the CPT-11 monotherapy group in the EPIC trial (3.3%, 2.4%, 25.4%, and 0.7%, respectively) [16]. In contrast, the incidence of grade ≥ 3 diarrhea in the present study (group A, 5.7%; group B, 0%) was lower than that in the EPIC trial (15.7%).

The ADRs observed in the present study were consistent with those reported in the phase I trials of NK012 that have been conducted in Japan [10] and the US [18] in patients with advanced solid carcinoma, in whom hemotoxicity and myelosuppression, particularly neutropenia, have been noted. In group A, the incidence of grade ≥ 3 neutropenia, classified as an abnormal laboratory value, and grade 3 febrile neutropenia, classified as a subjective or objective symptom, was 98.1% and 26.4%, respectively. Moreover, the incidence of grade 4 neutropenia in group A was 81.1%. Although myelosuppression was the main reason for dose reduction and treatment postponement, ADRs related to neutropenia could be controlled by appropriate treatment with G-CSF, antibiotics, or treatment postponement. The results of the present study suggest that a regimen of NK012 28 mg/m^2^ q3w may be excessive for patients with a history of oxaliplatin-based treatment. Of the 48 patients who received 2 or more cycles, 32 patients (66.7%) had their doses reduced to 22 mg/m^2^, while 8 (28.6%) of the 28 patients who received 3 or more cycles needed to have their doses reduced to < 22 mg/m^2^. Based on these results, further investigation regarding the appropriate dosage and administration of NK012 in the treatment of colorectal cancer is necessary.

Diarrhea is one of the most important complications of cancer therapy. It is thought that either the CPT-11 itself, or SN-38, causes diarrhea because of high levels of exposure in the intestines [19]. It has also been suggested that camptothecin derivatives such as CPT-11 have a carbamoyl structure that exerts adverse cholinergic effects, leading to cholinergic symptoms including diarrhea [20]. CPT-11, SN-38, and SN-38 glucuronide are excreted in the intestine, and some of this is recycled through enterohepatic circulation; thus, damage to the intestinal epithelial cells due to the intestinal excretion of CPT-11 and SN-38 may be involved [21–23]. It has also been suggested that CTP-11 is converted into SN-38 locally in the intestine by carboxylesterases [24]. In addition, it has been reported that SN-38 glucuronide is reconverted into SN-38 via β-glucuronidase in the intestinal microflora in rats [22, 23]. These various mechanisms of action can lead to regional increase in the SN-38 concentration in the intestine, which may explain the intestinal toxicity that is caused by CPT-11. Because NK012, on the other hand, does not require conversion by carboxylesterase, it is not likely to damage the intestinal epithelial cells during metabolism or cause a regional increase in the SN-38 concentration that results in intestinal toxicity.

Patients with UGT1A1*6/*28 or UGT1A1*6/*6 are known to have a reduced metabolism of SN-38 [25–27]. Therefore, the authors thought that the dose of NK012 in group B should be set lower than the dose of 28 mg/m^2^ used in group A to prevent serious ADRs from occurring. In the phase I trials in Japan and the US, no recommended dose for patients with UGT1A1*6/*28 or UGT1A1*6/*6 has yet been determined due to the insufficient number of subjects. However, in a US phase I trial enrolling patients whose pharmacokinetics are thought to be similar to Japanese, NK012 was tolerated in seven patients at doses of 4.5–18.5 mg/m^2^. Based on this result, the authors selected 18 mg/m^2^ as the initial dose for group B since it was within the maximum dose in the previous study. Because only five patients were enrolled in group B, a satisfactory analysis was difficult. Further studies enrolling a larger number of patients are necessary to investigate the relationship between *UGT1A1* gene polymorphisms and the safety (and efficacy) of NK012 so as to determine an optimum initial dose for this genetic population.

In conclusion, in patients with unresectable metastatic colorectal cancer who had previously received oxaliplatin-based therapy, the efficacy of NK012 therapy (repeated 3-week cycles) was similar to that of CPT-11 monotherapy [16]; however, since 56.6% of patients achieved DCR and some achieved PFS > 6 months, the authors have concluded that the treatment resulted in disease stabilization. The results also suggest that the incidence of serious diarrhea, which is a major problem in the clinical application of irinotecan hydrochloride, was lower than that with CPT-11. Based on the incidence and severity of grade ≥ 3 neutropenia and febrile neutropenia, the authors believe that an initial dose of NK012 28 mg/m^2^ may be excessive in patients with colorectal cancer who have previously received oxaliplatin-based therapy, and that the initial dose should be reduced in the next study. Further studies are necessary to determine the optimal dose of NK012 so as to enhance efficacy and safety. Currently, combination therapies of NK012 with other anticancer drugs in non-clinical studies for several types of tumors including colorectal cancer are yielding improved efficacy [9, 28–31], and further investigations of various combination therapies are expected.
